# COVID-19, sports, and myocardial consequences

**DOI:** 10.1007/s12471-020-01499-7

**Published:** 2020-10-08

**Authors:** H. T. Jørstad, J. J. Piek

**Affiliations:** Department of Cardiology, Heart Center, Amsterdam Cardiovascular Sciences, Amsterdam UMC, University of Amsterdam, Amsterdam, The Netherlands

During the first months of the severe acute respiratory syndrome coronavirus 2 (SARS-CoV-2) pandemic, the published data led to the assumption that cardiac involvement is correlated with the severity of the clinical course of the coronavirus disease 2019 (COVID-19). Major efforts were undertaken to predict who would be at risk for severe COVID-19, successfully identifying factors such as coronary artery disease, congestive heart failure, and obstructive lung disease [1]. In many ways, these factors characterise the opposite of what constitutes the healthy and relatively young populations of (elite) athletes and highly active individuals [2]. Yet, these are the very individuals—young and with a clinical presentation of SARS-CoV‑2 infection ranging from asymptomatic to mild—in whom increasingly alarming cardiac findings are being reported.

Initially, Puntmann et al. described cardiac magnetic resonance (CMR) imaging consistent with cardiac involvement 2 months after COVID-19 in upwards of two-thirds of patients [3]. This involvement was also observed in asymptomatic individuals and patients with a mild course of COVID-19. Following this, CMR imaging consistent with subclinical myocarditis was reported in 4 of 26 (15%) college athletes [4]. Around the same time, a 27-year-old professional basketball player was the first reported case of an elite athlete with COVID-19 associated sudden cardiac death (SCD) [5]. While possibly an isolated case, reports of increases in out-of-hospital cardiac arrests and SCD in regions with high rates of COVID-19 have emerged [6]. Therefore, a time-out to consider the ramifications of these findings seems more than warranted [7].

Why does this impact the whole field of cardiology? Physical exercise and sports form the preventive cornerstone for the majority of cardiovascular pathology. Yet, with no known baseline of myocardial involvement after regular viral infections, and no established prevalence for myocardial scar in athletes, limited hard data is available to formulate cardiac-specific advice for a return to sports after COVID-19. In light of this, two extreme scenarios can be hypothesised. One extreme would be to assume the worst and issue blanket advice to refrain from sports for a duration of 3–6 months, as one would after myocarditis. The consequences of this approach could potentially have severe negative effects on public health, and have a major impact on organised and competitive sports. The other extreme would be to adhere to the current position papers, which generally recommend pragmatic, non-sensitive screening, and a return to sports after approximately 2 weeks depending on disease severity [8, 9]. With this approach, myocardial involvement is potentially missed, and individuals who develop myocardial scars are only identified retrospectively. Both these alternatives are highly unattractive, and unfeasible in the long term.

Therefore, a concerted effort to elucidate the interaction between SARS-CoV‑2, the myocardium, and sports should be prioritised by scientific policy makers, professional societies, and funding bodies. Incidence and prevalence numbers are currently based on a limited number of studies, and need replication in larger cohorts. Simultaneously, diagnostic tests or predictors other than CMR that help clinicians identify individuals with subclinical myocarditis or at risk for developing myocardial fibrosis would be of significant added value in daily practice. In individuals with documented SARS-CoV‑2 myocardial sequalae, long-term follow-up should be performed to quantify potential risks of arrythmia and deterioration of myocardial function. Finally, based on the emerging data, multi-disciplinary collaborations are needed to reach a consensus on clear and practical advice regarding sports and exercise for individuals who have recovered from SARS-CoV‑2 infection.

The infrastructure of sports and sports cardiology in the Netherlands is uniquely suited to contribute. First, the NOC*NSF (https://nocnsf.nl/en) maintains a highly organised sports network for both elite and recreational sports, allowing for easy identification of athletes who have recovered from COVID-19. Second, specialised sports physicians form a first line of contact for athletes seeking medical advice, and can facilitate study recruitment. Third, ELITE (Evaluation of Lifetime participation in Intensive Top-level sports and Exercise), a collaboration between the University Medical Centres in Amsterdam and Utrecht and the NOC*NSF, includes a large number of pre-COVID-19 CMR scans in athletes, ideally suited for prospective investigations. Finally, with an active Netherlands Society of Cardiology Sports Section, cardiologists and allied professionals are poised to make a significant contribution to safety in sports after COVID-19. Team effort and collaboration are required, but the ball is now in our court—it is time to aim, shoot, and score.

## References

[1] Zhou F, Yu T, Du R, Fan G, Liu Y, Liu Z (2020). Clinical course and risk factors for mortality of adult inpatients with COVID-19 in Wuhan, China: a retrospective cohort study. Lancet.

[2] D’Ascenzi F, Caselli S, Alvino F, Digiacinto B, Lemme E, Piepoli M (2019). Cardiovascular risk profile in Olympic athletes: an unexpected and underestimated risk scenario. Br J Sports Med.

[3] Puntmann VO, Carerj ML, Wieters I, Fahim M, Arendt C, Hoffmann J (2020). Outcomes of cardiovascular magnetic resonance imaging in patients recently recovered from coronavirus disease 2019 (COVID-19). JAMA Cardiol.

[4] Rajpal S, Tong MS, Borchers J, Zareba KM, Obarski TP, Simonetti OP (2020). Cardiovascular magnetic resonance findings in competitive athletes recovering from COVID-19 infection. JAMA Cardiol.

[5] MailOnline. Ex-Florida State basketball player Michael Ojo, 27, dies ‘from a heart attack while training in Serbia after recently recovering from coronavirus. 2020. https://www.dailymail.co.uk/news/article-8604575/Basketball-player-dies-training-Serbia.html. Accessed 28 Sept 2020, pp 1–83.

[6] Baldi E, Sechi GM, Mare C, Canevari F, Brancaglione A, Primi R (2020). Out-of-hospital cardiac arrest during the Covid-19 outbreak in Italy. N Engl J Med.

[7] Topol EJ (2020). COVID-19 can affect the heart. Science.

[8] Verwoert GC, de Vries ST, Bijsterveld N, Willems AR, van der Borgh R, Jongman JK (2020). Return to sports after COVID-19: a position paper from the Dutch Sports Cardiology Section of the Netherlands Society of Cardiology. Neth Heart J.

[9] Wilson MG, Hull JH, Rogers J, Pollock N, Dodd M, Haines J (2020). Cardiorespiratory considerations for return-to-play in elite athletes after COVID-19 infection: a practical guide for sport and exercise medicine physicians. Br J Sports Med.

